# Anesthetic effect of articaine and lidocaine in tooth extraction

**DOI:** 10.6026/973206300210297

**Published:** 2025-03-31

**Authors:** Abhigyan Manas, Archana H Lanje (Misurya), Savita Singh, Jagadeesh K.N, Subham Patra, Prasanna T.R, Santosh T, Vaibhav Awinashe

**Affiliations:** 1 Department of Oral & Maxillofacial Surgery, Faculty of Dental Sciences, Uttar Pradesh University of Medical Sciences, Saifai, Etawah, Uttar Pradesh, India; 2 Department of Dentistry, MLB Medical College, Jhansi, Uttar Pradesh, India; 3 Oral and Maxillofacial Surgery Clinic, T-265 B, Chirag Delhi, New Delhi, India; 4 Department of Maxillofacial Prosthodontics & Implantology, Department of Dentistry, Siddaganga Medical College & Research Institute, Tumakur - 572102, Karnataka, India; 5 Intern, Kalinga Institute of Dental Sciences, Bhubaneswar, Odisha, India; 6 Department of Dentistry, Siddaganga Medical College & Research Institute, Tumakur - 572102, Karnataka, India; 7 Department of Oral Pathology & Microbiology, Government Dental College, Dibrugarh, Assam, India; 8 Department of Prosthodontics, College of Dentistry, Qassim University, Kingdom of Saudi Arabia

**Keywords:** Articaine, efficacy, lidocaine, local anaesthesia, pain management

## Abstract

The safety and efficiency of 4% articaine compared to 2% lidocaine for tooth extraction is of interest to dentists. Hence, a total of 30 patients requiring premolar tooth extraction for orthodontics reason were randomly divided into 2 groups namely (1) articaine and (2) lidocaine. Parameters such as onset of action, duration of anaesthesia and need to re-anesthetize at the surgical zone are evaluated. Pain evaluation was done with visual analog scale (VAS). Data shows that 4% articaine had a shorter onset of action and longer duration of anaesthesia compared to lidocaine.

## Background:

Performing painless tooth extractions is still a major concern for both patients and dental surgeons [1]. Since local anaesthesia is believed to be the safest and most efficient for preventing and managing pain in patients having oral surgery, it is essential for pain management in dentistry [2]. Tooth extractions can be performed for a variety of reasons, including advanced periodontal diseases, irreversible dental pulpitis and orthodontics. Because of the unpleasant experience of having their teeth extracted or those of others extracted in the past, many people think that tooth extraction as one of the most dreaded surgeries [3]. The solutions for pain management during treatments are provided by local anaesthesia [2]. The potency, latency and duration of the local anaesthetic drug are the primary determinants in its selection [4]. Because of its pharmacokinetic properties and minimal toxicity when compared to other ester-type anaesthetics, lidocaine is the most commonly utilised local anaesthetic for pain management in dentistry [5]. Because of its brief duration of action, alternative LA is being studied. The best local anaesthetic (LA) drug that can generate a quicker onset and longer duration is still being in search by dental researchers [1]. Articaine, a more recent amide local anaesthetic has been utilised in clinical dentistry for the past 20 years and is a safe and efficient anaesthetic. Due to the presence of a thiophenic ring with additional ester group in substitution with aromatic ring, its chemical structure has different properties as compared with other local aesthetic. More lipid solubility is made possible by the thiophene ring, which makes it easier to diffuse across the lipid-rich neuronal membrane and reach target receptors [1-6]. As a result, articaine has higher intrinsic potency (1.5 times higher than lidocaine), improved lipid solubility and higher plasma protein binding (around 95%) [5]. Compared to other anaesthetics, articaine has a higher diffusion rate and is more capable of diffusing in both soft and hard tissue [3, 7]. It eliminates the need for a painful palatal injection by providing soft tissue anaesthesia on the palatal side through buccal infiltration in the maxilla [3]. Therefore, it is of interest to compare the effectiveness and safety of 4% articaine versus 2% lidocaine in tooth extraction.

## Materials and Methods:

Following institutional ethics committee permission and participant informed consent, the current study was conducted in the Department of Oral and Maxillofacial Surgery. Participants in the trial had to be ASA class I patients between the ages of 18 and 30, free of systemic diseases and in good oral and periodontal health. Total 30 patients requiring maxillary premolar tooth extraction for orthodontics reason were randomly divided into 2 groups (15 each) as; Group-I-Articaine and Group II-lidocaine. The study employed either lidocaine HCl 2% with epinephrine 1:100,000 injection (lidocaine hydrochloride and epinephrine injection USP, Novocol, India) or Septodont Septanest: 1:100,000-4% articaine with epinephrine (Septanest, SeptodontInc, France). Single trained operator used one of the local aesthetic agents to accomplish orthodontic extraction of premolars under aseptic settings. Premolar teeth were surgically extracted according to a set procedure. A total of 3.6 millilitres of anaesthetic solution were given to each patient. After extraction patients were advised to take analgesics. The total volume of anaesthetic solution used during the procedure, the anaesthetic agent's onset of action, the length of anaesthesia and postoperative analgesia, the kind and severity of adverse reactions and the necessity of re-anesthetizing the surgical area were all factors that were assessed for the effectiveness of both LA agents. Throughout the extraction procedure, questions about pain were asked of each patient on a regular basis. A VAS scale with a reading range of 0 to 100 was used to measure the amount of pain experienced both during and after the extraction. A pain VAS score of 0 to 100 was considered as mild to severe intensity. The obtained data was statistically evaluated using SPSS statistical software version 23.0 with p>0.05.

## Results:

Table 1 shows that compared to lidocaine, articaine had a statistically significant faster onset of action, a longer duration of aesthetic action and a lower need for re-anaesthesia. Additionally, we saw that while all of the Group II patients had palatal infiltrations, but none of the Group I patients required palatal infiltration. Group I experienced post-operative analgesia for a longer period of time than Group II. Table 2 indicates that, intra operative and post-operative pain score was lesser in group I compared to Group II but it was statically not significant.

## Discussion:

Every surgeon's primary objective is to manage pain [7]. A local anaesthetic is frequently used to treat dental pain. Articaine is a recently developed local anaesthetic drug due to its relative potency and safety [8]. The onset of an anaesthetic solution is influenced by a number of factors, including the drug's intrinsic qualities, method and pKa value; the lower the pKa, the shorter the latency. Articaine has a shorter latent period than lidocaine because it has a lower pKa value [9]. The degree of protein binding, the injection site and the amount of vasoconstrictor added to the solution all affect how long anaesthesia lasts. Out of all the amides, articaine has the highest protein-binding values [6, 10]. Current research has shown that both the local anaesthetics were effective in providing sufficient anaesthesia during tooth extraction. However, we found that articaine had faster onset, longer duration of action, requires lesser volume of anaesthesia compared to lidocaine. According to Sisk, the anaesthesia used during the procedure was successfully provided using a 2% lidocaine to epinephrine 1:100,000 mixtures [11]. For mandibular posterior teeth, Robertson *et al.* observed that articaine administered by local infiltration provided good analgesia [12]. While extracting premolars, Jaiswal *et al.* examined the anaesthetic efficacy of lignocaine and articaine. They came to the conclusion that articaine was a viable substitute for lidocaine [2]. In order to remove impacted mandibular third teeth, Mittal *et al.* assessed the safety and effectiveness of 4% articaine vs 2% lidocaine. They came to the conclusion that 4% articaine had a longer duration of anaesthesia and a shorter onset of action than 2% lidocaine [4]. Majid *et al.* came to the conclusion that, in tooth extraction, lidocaine had anaesthetic adequacy that was statistically lower than that of 4% articaine [13]. According to Gholami *et al.* articaine may be a good substitute for lidocaine in the treatment of painful palatal infiltration during maxillary tooth extraction [14]. These outcomes are consistent with what we found. According to Rebolledo *et al.* 4% articaine performs better clinically than 2% lidocaine [5]. According to Kumar *et al.* articaine can be used as a substitute for lignocaine and is clinically more effective than lignocaine [7]. These results are related to our findings. Because articaine produced a concentration of active anaesthetic molecules at the injection site that was twice as high as lignocaine, studies have shown that it is efficacious at lower volumes than lignocaine [3]. According to Cowan, articaine created a large concentration of active anaesthetic molecules locally, which resulted in a rapid and persistent action when combined with a vasoconstrictor [8]. According to Gazal, articaine is a fast-acting anaesthetic that works similarly to mepivacaine in infiltrative procedures for extracting maxillary teeth [1]. A 100-mm VAS was used in this study to subjectively assess the pain experienced during and after surgery. Numerous publications have employed the VAS to assess pain subjectively [4, 15]. According to Boonsiriseth *et al.* patients reported less intra-operative pain and greater analgesia during the surgery with 4% lidocaine than with 4% articaine; nevertheless, the difference was not clinically significant [16]. According to Kumar *et al.* there is no difference in the degree of discomfort or the time at which anaesthesia begins, but the length of anaesthesia is greater with Articaine than with Lignocaine [17]. According to a study by Zhang *et al.* 4% articaine with 1:100000 epinephrine has a higher anaesthetic efficacy than lidocaine [18]. We found better result with articaine compared to lidocaine which is in accordance to many researchers findings mentioned above, hence articaine can be suggested as an alternative to lidocaine in pain management and tooth extraction in dentistry. Further studies are needed to validate the results.

## Conclusion:

Lignocaine is regarded as the gold standard for local anaesthesia. We show that 4% articaine has a longer half-life and a faster start of action than lignocaine for an alternative consideration.

## Figures and Tables

**Table 1:** Assessment of onset of action, duration of anaesthesia and postoperative analgesia

**Parameter**	**Group I (mean ± SD)Articaine**	**Group II (mean ± SD)Lidocaine**	p
Onset of anaesthesia (min)	1.02±2.13	2.47±2.64	0.001
Duration of anaesthesia (min)	85.42±2.34	43.55±2.42	0.021
Need of palatal injection	0	30	0.001
Volume of Drug required (ml)	1.21±0.24	1.92±0.25	
Duration of post-operative analgesia (min)	218±4.24	115±2.54	0.001

**Table 2:** Evaluation of pain score using VAS

**Parameters**	**Group I (mean ± SD)**	**Group II (mean ± SD)**	**p**
Intra operative pain score (mm)	4.17±3.64	10.43±4.23	0.324
Post-operative pain score (mm)	12.03±2.34	13.78±3.24	0.432
